# Association of E/E′ and NT-proBNP with Renal Function in Patients with Essential Hypertension

**DOI:** 10.1371/journal.pone.0054513

**Published:** 2013-01-28

**Authors:** Yan Yang, Yan Wang, Zhong-wei Shi, Ding-liang Zhu, Ping-jin Gao

**Affiliations:** 1 State Key Laboratory of Medical Genomics, Shanghai Key Laboratory of Vascular Biology, Department of Hypertension, Shanghai Institute of Hypertension, Ruijin Hospital, Shanghai Jiaotong University School of Medicine, Shanghai, China; 2 Department of Cardiology, Ruijin Hospital, Shanghai Jiaotong University School of Medicine, Shanghai, China; 3 Laboratory of Vascular Biology, Institute of Health Sciences, Shanghai Institutes for Biological Sciences, Chinese Academy of Sciences, Shanghai, China; The University of Texas Health Science Center, United States of America

## Abstract

**Objectives:**

To evaluate the association of left ventricular (LV) diastolic function and N-terminal pro-brain natriuretic peptide (NT-proBNP) with renal function in essential hypertension.

**Methods:**

LV diastolic function was estimated by the ratio of early diastolic velocities (E) from transmitral inflow to early diastolic velocities (E′) of tissue Doppler at mitral annulus (septal corner); NT-proBNP was measured in 207 hypertensive patients (mean age 56±14 years). The subjects were classified into 3 groups: E/E′≤10 group (n = 48), 10<E/E′≤15 group (n = 109) and E/E′>15 group (n = 50). The renal function was estimated by glomerular filtration rate (GFR) with ^99m^Tc-DTPA. GFR from 30 to 59 ml/min/1.73 m^2^ was defined as Stage 3 chronic kidney disease (CKD). GFR was also estimated using the modified MDRD equation. Albuminuria was defined by urinary albumin/creatinine ratio (UACR).

**Results:**

GFR was lower and UACR was higher in E/E′ >15 group than in 10< E/E′ ≤15 group or E/E′ ≤10 group (*p*<0.0001), GFR was significantly negative and UACR was positive correlated with E/E′ and NT-proBNP (*p*<0.0001). In multivariate stepwise linear analysis, GFR had significant correlation with age (*p* = 0.001), gender (*p* = 0.003), E/E′ (*p* = 0.03), lgNT-proBNP (p = 0.001) and lgUACR (*p* = 0.01), while eGFR had no significant correlation with E/E′ or lgNT-proBNP. Multivariate logistic regression analysis, adjusted for potential confounding factors, showed that participants in E/E′>15 group were more likely to have Stage 3 CKD compared with those in E/E′≤10 group with an adjusted odds ratio of 8.31 (*p* = 0.0036).

**Conclusions:**

LV diastolic function, assessed with E/E′ and NT-proBNP is associated with renal function in essential hypertension.

## Introduction

Chronic renal dysfunction has a negative effect on cardiac function [1], [2] and patients with chronic kidney disease (CKD) frequently display anomalies of left ventricular (LV) structure and function [3], [4]. Both left ventricular systolic and diastolic dysfunction are commonly observed in end-stage renal disease (ESRD), may be associated with subsequent development of cardiac failure and mortality [5], [6]. LV diastolic dysfunction usually precedes LV systolic dysfunction [7] and is associated with morbidity and mortality in chronically dialyzed patients or CKD patients [8]. Increased Urinary albumin/creatinine ratio (UACR) may reflect subclinical disease in renal dysfunction and was reported to predict cardiovascular death in hypertension [9]. Plasma concentrations of N-terminal pro-brain natriuretic peptide (NT-proBNP) increase with decompensated congestive heart failure (CHF) [10] or degree of LV diastolic function [11], which are also found to be elevated with mortality in ESRD [12]. Statement on diagnosis of heart failure with normal LV ejection fraction from the Heart Failure and Echocardiography Associations of the European Society of Cardiology, suggests that NT-proBNP can be used as a biomarker for heart failure [13].

Tissue Doppler imaging (TDI) is a new method for assessing left ventricular systolic and diastolic function in cardiac diseases. Diagnostic evidence of LV diastolic dysfunction can be obtained non-invasively by TDI (E/E′>15) according to the European Society of Cardiology [13]. Therefore, to evaluate LV diastolic function, the ratio of peak early transmitral flow velocity (E) to tissue Doppler early peak diastolic annular (E′), was calculated.

Despite extensive knowledge about cardiac structure and function in patients with ESRD [7], [8], the relationship between mild reductions in renal function and cardiac morphology and function in early hypertension has been less clear. The purpose of this study was to evaluate whether LV diastolic function is correlated with renal function, and to determine the association of E/E′ and NT-proBNP with glomerular filtration rate (GFR) measured with technetium-99m-(^99m^Tc) diethylenetriaminepentaacetic acid (DTPA) in essential hypertension.

## Methods

### Subjects

Two hundred and eighty patients were screened for this study, among them, 49 patients due to secondary hypertension, 10 patients due to suboptimal images and 14 patients due to absent biomarker results were excluded. A total of 207 essential hypertensive patients (mean age 56±14; 59% male) were enrolled into the study consecutively from the hypertension department or clinic in Ruijin hospital from January 2009 to January 2011. Prior informed consent was obtained from all patients for participation in the study. All the participants provide their written informed consent to participate in this study. This study was approved by the Ethics Committee of Ruijin Hospital.

The inclusion criterion was a diagnosis of essential hypertension according to 2005 Chinese guidelines for the prevention and treatment of patients with hypertension [14]. Patients with atrial fibrillation, systolic left ventricular dysfunction, chronic heart failure, severe arrhythmia, myocardial infarction, cardiomyopathy, valvular disorders, secondary hypertension or GFR below 30 ml/min/1.73 m^2^ were excluded from the study. Systolic left ventricular dysfunction is defined as ejection fraction less than 50%.

### Echocardiography

Comprehensive transthoracic echocardiography was performed using a cardiac ultrasound system (HD11XE, Philips, USA). LV diameters, septal and posterior wall thickness were measured according to the guidelines of the American Society of Echocardiography [15]. LV mass in grams was calculated from M-mode echocardiograms according to the formula described by Devereux *et al* [16]. LV mass was indexed to body surface area as LV mass index (LVMI) in g/m^2^ body surface area. Left atrial volume was indexed by body surface area as LA volume index (LAVI) in g/m^2^ body surface area.

The peak early (E) and late (A) transmitral flow velocities, the ratio of early to late peak velocities (E/A), deceleration time of E velocity and isovolumic relaxation time (IVRT) were measured. The mitral flow velocity was obtained from apical 4-chamber view by placing a pulsed-wave (PW) Doppler sample volume between mitral leaflet tips during diastole. The curve of continues wave Doppler was put between the LV out flow and inflow tract and recording a trace of LV out flow tract and inflow waves from the apical 5-chamber view. The IVRT measurement was estimated from the cessation of the aortic flow and the onset of transmitral inflow.

Tissue Doppler imaging (TDI) was performed by means of mitral annulus in four-chamber view. The early peak diastolic annular (E′) was determined from the TDI recordings. The ratio of peak early (E) to tissue Doppler early diastolic (E′) was calculated.

According to the statement of the European Society of Cardiology [13], we defined LV diastolic dysfunction as (a) normal or mildly reduced LV systolic function: LVEF>50%; and (b) E/E′>15.

To calculate the intra-observer variability, the parameters were reassessed in the first 20 patients by the same observer without reviewing the patients’ previous reports, 4 weeks after the first set of the evaluation.

### Plasma NT-proBNP

Plasma NT-proBNP levels were determined using the electrochemiluminescence immunoassay “ECLIA” on Elecsys and cobas e immunoassay analyzers (Roche Diagnostics, Basel, Switzerland).

### Assessment of Renal Function

Renal function was measured by GFR following injection of ^99m^Tc-DTPA, an agent commonly used for renal scintigraphy. GFR from 30 to 59 ml/min/1.73 m^2^ was defined as Stage 3 CKD. GFR was also estimated using the modified MDRD equation for Chinese patients with chronic kidney disease [17] in all the patients.

UACR was measured to identify subclinical renal damage. Urine albumin was measured quantitatively with an immunoephelometry test (Siemens BN-TROStec, Germany) and creatinine was assessed quantitatively with an enzymatic colorimetric test (Hitachi automatic analyzer 7600-020, Japan). UACR was calculated using the following formula: [urine albumin(mg/l)/urine creatinine(g/l)].

### Statistical Analysis

Data were stored and analyzed using the SPSS 13.0 statistical package (SPSS Inc, Chicago, IL). All results are presented as mean values ± SD. Because NT-proBNP and UACR levels were not normally distributed, lgNT-proBNP and lgUACR were used in correlations and regression models. The difference of the intercept of the linear regression line between 2 groups was analyzed using analysis of covariance. Differences between studied groups were compared by one-way ANOVA, and differences with respect to categorized data by χ2 test. Multivariable adjusted test for trend was employed to assess the association between lgUACR and E/E′ or lgNT-proBNP categories after adjusting for baseline clinical characteristics. Linear regression was performed for the univariate model of GFR, eGFR and serum creatinine. Multivariable linear regression models for the relationship between continuous variables and GFR or eGFR category adjusted for age, gender, the use of ACE-I or ARB drugs, blood pressure, heart rate, and covariates that differed significantly by GFR or eGFR category with *p* value of 0.05 or less: LVMI, LAVI, E/A, deceleration time, IVRT, E/E′, lgNT-proBNP and lgUACR. In the multivariable logistic regression, models, we adjusted for the potential confounding factors including age, sex, body mass index, prevalence of diabetes, the use of ACE-I or ARB drugs, 24 h mean systolic blood pressure, LVMI, LAVI, E/A, deceleration time, IVRT, lgNT-proBNP and lgUACR to evaluate the association of E/E′ with Stage 3 CKD. A *p* value of <0.05 was defined as statistically significant, and a *p* value of <0.01 as highly significant.

## Results

### Patient Characteristics

The subjects were classified into 3 groups based on left ventricular diastolic function: group 1 with normal LV diastolic function of E/E′≤10 (n = 48, 23.2%); group 2 with suspected LV diastolic dysfunction of 10<E/E′≤15 (n = 109, 52.7%); group 3 with LV diastolic dysfunction of E/E′>15 (n = 50, 24.2%). The variation coefficient of LVMI, LAVI, E/A, deceleration time, IVRT and E/E′ was 7.2%, 6.1%, 8.4%, 8.3%, 6.2% and 6.3% respectively.

Anthropometric, metabolic, hemodynamic, and echocardiographic characteristics, as well as the medical history of the study patients are included in Table 1. Patients in group 3 (E/E′>15) were older and characterized by higher 24-h mean systolic blood pressure (SBP), lower 24-h mean diastolic blood pressure (DBP), higher 24-h mean pulse pressure and slower 24-h mean heart rate (HR) than in group 1 or 2. There was no difference in gender, incidence of diabetes, fasting glucose, serum cholesterol, triglycerides, high sensitivity C-creative protein, serum creatinine, left ventricular systolic function, E/A and DT in these 3 groups. In group 3 patients, LAVI (*p*<0.0001), LVMI (*p*<0.0001), IVRT (*p* = 0.0003), lgNT-proBNP (*p*<0.0001) and lgUACR (*p*<0.0001) were significantly higher, while GFR (*p*<0.0001) and eGFR (*p* = 0.0002) were significantly lower (*p* = 0.0002) than in group 1 or 2. Patients in group 2 were older and had higher BMI, LVMI, lgNT-proBNP than those in group 1. There was no difference in the using of antihypertensive medicine in the 3 groups.

**Table 1:** Clinical characteristics.

Variables	Group 1 E/E′≤10(n = 48)	Group 210<E/E′≤15(n = 109)	Group 3E/E′>15 (n = 50)	P value
Age (years)	47.8±15.5	54.9±11.8^a^	64.4±12.4a,b	<0.0001
Male, n (%)	31 (65)	65 (60)	25(50)	0.06
Body mass index (kg/m^2^)	24.6±3.7	25.9±3.3^a^	24.9±3.2	0.04
24-h mean SBP (mmHg)	126±13	130±16	135±16^a^	0.03
24-h mean DBP (mmHg)	80±10	81±11	75±9a,b	0.004
24-h mean PP (mmHg)	46±8	49±12	60±13a,b	<0.0001
24-h mean HR (bpm)	74±9	72±9	67±7a,b	0.002
Diabetes, n (%)	9 (19)	21 (19)	12 (24)	0.75
Serum TC (mg/dl)	189±39	182±43	186±31	0.75
Serum TG (mg/dl)	168±132	204±150	177±177	0.45
Fasting glucose (mg/dl)	97±16	99±20	99±31	0.86
lg NT-proBNP	1.3±0.5	1.5±0.5^a^	2.2±0.5a,b	<0.0001
hs-CRP (mg/l)	2.5±4.0	2.8±4.0	4.3±8.4	0.23
BUN (mg/dl)	6.6±1.7	7.3±2.1	8.4±5.2^a^	0.018
Serum creatinine (mg/dl)	0.88±0.14	0.95±0.28	1.03±0.40^a^	0.052
eGFR(ml/min/1.73 m^2^)	114±17	104±26	97±27a,b	0.0002
GFR(ml/min/1.73 m^2^)	88±18	82±21	65±20a,b	<0.0001
lg UACR	1.03±0.39	1.19±0.47	1.62±0.70a,b	<0.0001
Echocardiographic parameter				
LVMI (g/m^2^)	100±19	111±24^a^	134±37a,b	<0.0001
LV Shortening fraction (%)	37±4	37±5	37±4	0.97
LAVI (ml/m^2^)	20.9±4.6	22.4±3.9	26.6±5.2a,b	<0.0001
E/A	1.08±0.35	1.00±0.33^a^	0.97±0.34	0.68
Deceleration time (ms)	230±50	221±49	239±57^b^	0.12
IVRT (ms)	102±22	107±26	124±39a,b	0.0003
Anti-hypertensive drugs				
ACE-I, n (%)	13 (27)	30 (28)	17 (34)	0.67
ARB, n (%)	16 (33)	46 (42)	20 (40)	0.58
CCB, n (%)	36 (75)	82 (75)	38 (76)	0.99
Beta-Blocker, n (%)	12 (25)	30 (28)	21 (42)	0.12
Diuretic, n (%)	8 (17)	27 (25)	16 (32)	0.21

SBP: systolic blood pressure; DBP: diastolic blood pressure; PP: pulse pressure; HR: heart rate; TC: total cholesterol; TG: triglycerides; hs-CRP: high sensitivity C-creative protein; UACR: Urinary albumin creatinine ratio; ACE-I: medication with angiotensin-converting enzyme (ACE) inhibitor; ARB: medication with angiotensin receptor blocker; CCB: medication with calcium channel blocker; LVMI: left ventricular mass index; LAVI: left atrial volume index; IVRT: isovolumic relaxation time.

a Significant difference with group 1.

b Significant difference with group 2.

### Association between Renal Function and Left Ventricular Diastolic Function or Plasma NT-proBNP

As shown in Table 2, Pearson’s correlation analyses revealed that GFR was significantly correlated with age(*p*<0.0001), gender(*p* = 0.002), 24-h mean systolic blood pressure(*p* = 0.043), 24-h mean heart rate(*p* = 0.001), LVMI(*p*<0.0001), LAVI(*p*<0.0001), E/A(*p* = 0.003), deceleration time(*p* = 0.006), IVRT(*p*<0.0001), E/E′ (*p*<0.0001), lgNT-proBNP(*p*<0.0001) and lgUACR(*p*<0.0001). Of the 42 diabetes patients in our study, 19 patients were new-developed diabetes and 23 patients were with diabetes history. In all the 42 diabetes patients, GFR had no relationship with diabetes (*r* = −0.045, *p* = 0.54).

**Table 2:** Univariate and multiple stepwise linear model of GFR.

Variables	Univariate		Stepwise	
	r	P	Standardized β± SE	P
Age	−0.431	<0.0001	−0.30±0.22	0.001
Gender	0.217	0.002	0.22±3.24	0.003
Body mass index (kg/m^2^)	0.103	0.15		
Use of ACE-I or ARB drugs	0.052	0.29		
24-h mean SBP (mmHg)	−0.155	0.043		
24-h mean HR (bpm)	0.255	0.001		
Diabetes	−0.045	0.54		
LVMI (g/m2)	−0.325	<0.0001		
LAVI (ml/m2)	−0.305	<0.0001		
E/A	0.216	0.003		
Deceleration time (ms)	−0.198	0.006		
IVRT (ms)	−0.284	<0.0001		
E/E′	−0.374	<0.0001	−0.21±0.53	0.03
lg NT-proBNP	−0.463	<0.0001	−0.29±3.18	0.001
lg UACR	−0.347	<0.0001	−0.20±3.13	0.01

Abbreviations as in Table 1.

A multivariable stepwise linear regression analysis was performed to evaluate the independent determinants of GFR using age, gender, prevalence of diabetes, ACE-I or ARB usage, BMI, 24-h mean systolic blood pressure, 24-h mean heart rate, LVMI, LAVI, E/A, deceleration time, IVRT, E/E′, lgNT-proBNP and lgUACR. We found that GFR was significantly correlated with age (*p* = 0.001), gender (*p* = 0.003), E/E′ (*p* = 0.03), lgNT-proBNP (*p* = 0.001) and lgUACR (*p* = 0.01) in multivariate stepwise linear analysis (Table 2).

**Figure 1:**
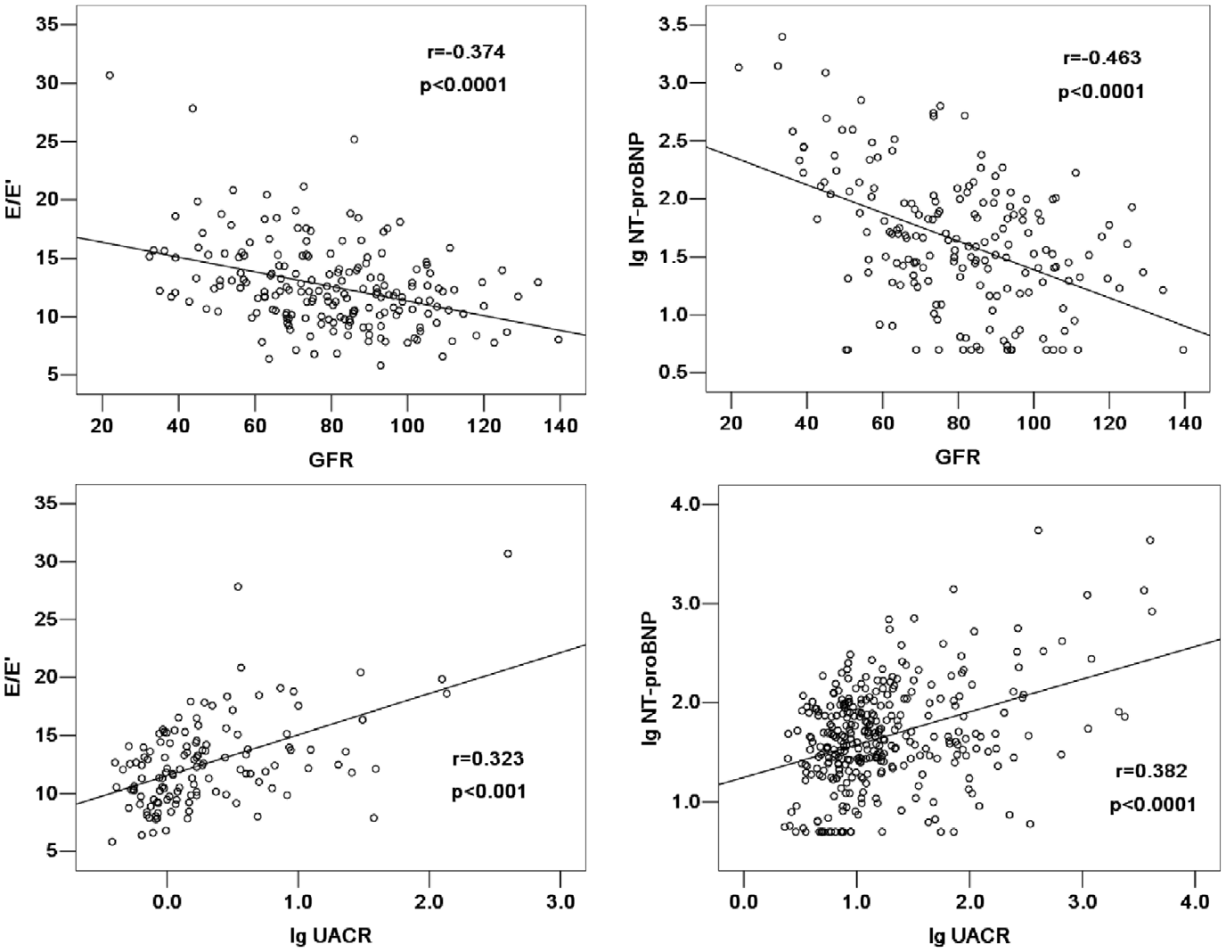
The relationship between GFR or UACR and E/E′ or NT-proBNP. GFR was significantly correlated with E/E′ and log-transformed plasma NT-proBNP, the correlation coefficient was −0.374 and −0.463 respectively (P<0.0001). Log-transformed UACR was also significantly correlated with E/E′ and log-transformed plasma NT-proBNP, the correlation coefficient was 0.323 and 0.382 respectively (P<0.0001).

Figure 1 showed the significant correlation between GFR and E/E′, as well as lgNT-proBNP (the correlation coefficient was −0.374 and −0.463 respectively, *p*<0.0001). Logarithmically transformed UACR was significantly correlated with E/E′ and lgNT-proBNP (the correlation coefficient was 0.323 and 0.382 respectively, *p*<0.0001). After adjustment for age, gender, diabetes prevalence, use of ACE-I or ARB drugs, GFR, 24-h mean systolic and diastolic blood pressure, 24-h mean heart rate, lgUACR was also significantly correlated with E/E′ (the correlation coefficient was 0.364, *p*<0.0001) and lgNT-proBNP (the correlation coefficient was 0.191, *p* = 0.044). E/E′ was significantly correlated with lgNT-proBNP, the correlation coefficient was 0.544 (*p*<0.001).

In all patients, GFR was estimated using the modified MDRD equation for Chinese patients with CKD [19] and was correlated with age(*p*<0.0001), gender(*p* = 0.007), 24-h mean heart rate(*p* = 0.001), LVMI(*p*<0.0001), LAVI(*p*<0.0001), deceleration time(*p* = 0.003), IVRT(*p*<0.0001), E/E′ (*p*<0.0001), lgNT-proBNP(*p*<0.0001) and lgUACR(*p*<0.0001) in Pearson’s correlation analysis (Table 3). In multivariate stepwise linear analysis, we found that eGFR was significantly correlated with age (*p*<0.0001), gender (*p* = 0.02), lgUACR (*p* = 0.005) and IVRT (*p* = 0.02), but had no significant correlation with E/E′ or lgNT-proBNP (Table 3). Serum creatinine was not associated with lgNT-proBNP or E/E′ in univariate analysis (data not shown).

**Table 3:** Univariate and multiple stepwise linear model of eGFR.

Variables	Univariate		Stepwise	
	r	P	Standardized β± SE	P
Age	−0.373	<0.0001	−0.33±0.13	<0.0001
Gender	0.190	0.007	0.18±3.62	0.02
Body mass index (kg/m^2^)	0.018	0.80	–	–
Use of ACE-I or ARB drugs	−0.059	0.41	–	–
24-h mean SBP (mmHg)	−0.134	0.07	–	–
24-h mean HR (bpm)	0.250	0.001	–	–
Diabetes	−0.059	0.40	–	–
LVMI (g/m2)	−0.383	<0.0001	–	–
LAVI (ml/m2)	−0.305	<0.0001	–	–
E/A	−0.133	0.06	–	–
Deceleration time (ms)	−0.207	0.003	–	–
IVRT (ms)	−0.335	<0.0001	−0.21±61.30	0.02
E/E′	−0.299	<0.0001	–	–
lg NT-proBNP	−0.331	<0.0001	–	–
lg UACR	−0.341	<0.0001	−0.22±3.36	0.005

Abbreviations as in Table 1.

**Figure 2:**
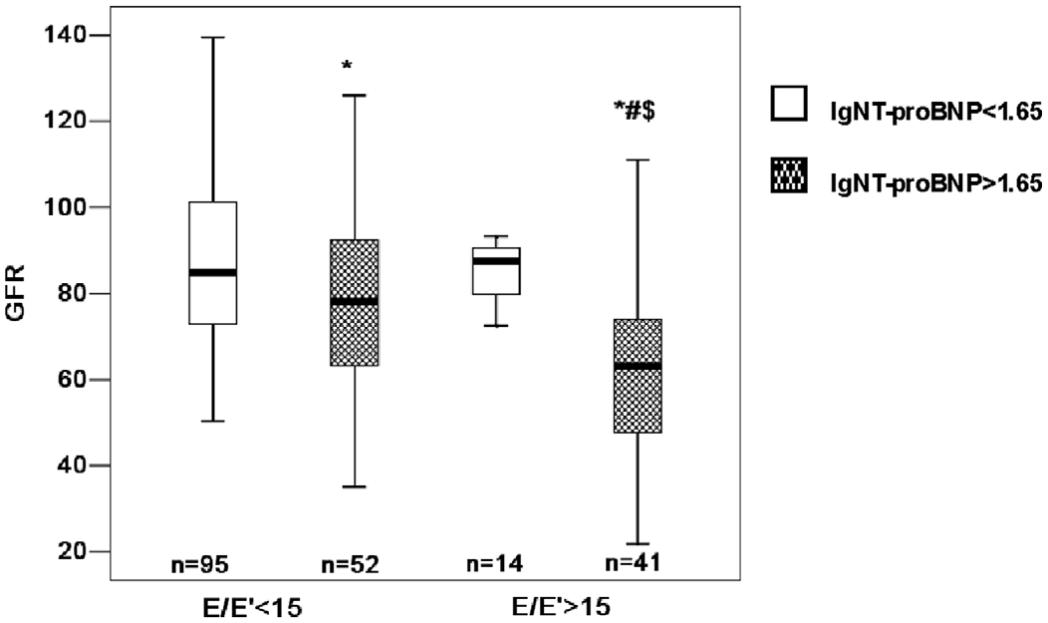
Association of E/E′ and NT-proBNP with GFR. (white box, lgNT-proBNP<1.65; shaded box, lgNT-proBNP>1.65) * P<0.01 vs lgNT-proBNP<1.65 and E/E′<15, # P<0.01 vs lgNT-proBNP>1.65 and E/E′<15, $ P<0.05 vs lgNT-proBNP<1.65 and E/E′>15.

The median of lgNT-proBNP was 1.65, so we divided the patients into 2 groups as shown in Figure 2: white box, lgNT-proBNP<1.65; shaded box, lgNT-proBNP>1.65. Patients with higher NT-proBNP had worse renal function than patients with normal NT-proBNP regardless of the presence or absence of LV diastolic dysfunction. GFR was even lower in the patients of E/E′>15 than in the patients of E/E′<15 with lgNT-proBNP>1.65. In patients with lgNT-proBNP>1.65, E/E′<15 was associated with higher GFR compared with E/E′>15 (GFR: 78±22 vs. 63±20, *p* = 0.001), while there was no difference in GFR between E/E′<15 group and E/E′>15 group in patients with lgNT-proBNP<1.65 (GFR: 87±19 vs. 85±9, *p* = 0.85).

### Association between E/E′ and Stage 3 CKD (Defined as 30<GFR<59 or 30<eGFR<59)

In the univariable logistic regression model, participants in group 3 were more likely to have Stage 3 CKD (defined as 30<GFR<59) compared with those in group 1 (odds ratio (OR): 6.46, 95% confidence intervals (95% CI) 2.33–19.91; *p* = 0.0003). The test for the trend was significant (*p* for trend <0.0001). After adjusting for age, gender, and body mass index, participants in group 3 were still more likely to have Stage 3 CKD compared with those in group 1 (adjusted OR: 6.01, 95% CI 1.95–18.45, *p* = 0.002). Further adjustments for other potential confounding factors including age, gender, body mass index, prevalence of diabetes, the use of angiotensin-converting enzyme inhibitor or angiotensin receptor blocker drugs, 24-h mean systolic blood pressure, LVMI, LAVI, E/A, deceleration time, IVRT, lgNT-proBNP and lgUACR did not materially change the magnitudes of the associations (adjusted OR for group 3 vs. group 1: 8.31, 95% CI 2.00–34.56, *p* = 0.004). The tests for the trend in the multivariable analyses were all significant (*p* for trend = 0.0006 and 0.002) (Table 4).

**Table 4:** Associations of E/E′ with Stage 3 CKD (defined as 30<GFR<59).

	Model 1		Model 2		Model 3	
	ORs(95% CI)	P	ORs(95% CI)	P	ORs(95% CI)	P
E/E′≤10	1 [reference]		1 [reference]		1 [reference]	
10<E/E′≤15	1.57[0.59–4.20]	0.36	1.64[0.58–4.59]	0.34	2.80[0.82–9.60]	0.09
E/E′>15	6.46[2.33–19.91]	0.0003	6.01[1.95–18.45]	0.002	8.31[2.00–34.56]	0.004
P for trend	<0.0001		0.0006		0.002	

Model 1: unadjusted.

Model 2: adjusted for age, gender, and body mass index.

Model 3: adjusted for all the potential confounding factors including age, gender, body mass index, prevalence of diabetes, use of ACE-I or ARB drugs, 24-h mean systolic blood pressure, LVMI, LAVI, E/A, deceleration time, IVRT, lgNT-proBNP and lgUACR.

Abbreviations as in Table 1.

No significant relationships between E/E′ and 30<eGFR<59 were observed (adjusted ORs for group 3 vs. group 1: 3.28, 95% CI 0.54–19.78; for group 2 vs. group 1: 2.76, 95% CI 0.51–15.08) (Table 5).

**Table 5:** Associations of E/E′ with Stage 3 CKD (defined as 30<eGFR<59).

	Model 1		Model 2		Model 3	
	ORs(95% CI)	P	ORs(95% CI)	P	ORs(95% CI)	P
E/E′≤10	1 [reference]		1 [reference]		1 [reference]	
10<E/E′≤15	2.07[0.43–9.96]	0.36	2.14[0.42–10.92]	0.35	2.76[0.508–15.08]	0.66
E/E′>15	3.744[0.74–19.02]	0.11	2.59[0.45–14.95]	0.28	3.28[0.54–19.78]	0.23
P for trend	0.08		0.31		0.21	

Model 1: unadjusted.

Model 2: adjusted for age, gender, and body mass index.

Model 3: adjusted for all the potential confounding factors including age, gender, prevalence of diabetes, use of ACE-I or ARB drugs, body mass index, 24-h mean systolic blood pressure, LVMI, LAVI, E/A, deceleration time, IVRT, lgNT-proBNP and lgUACR.

Abbreviations as in Table 1.

## Discussion

The current study investigated the relationship between LV diastolic function and renal function in early essential hypertension. We found that parameters of LV diastolic function including E/E′ and NT-proBNP levels were associated with renal impairment, manifested as both albuminuria and mild reductions in GFR. Using the reference technique of ^99m^Tc-DTPA clearance to measure GFR, precise and reliable relationships were found between cardiac and renal status, even in patients displaying only mild to modest declines in GFR.

In the present study, we used TDI to assess diastolic function of the left ventricle. By using the combination of TDI of mitral annulus and mitral inflow velocity from conventional Doppler (E/E′), which is relatively independent of loading conditions, was superior to conventional blood flow Doppler echocardiography indices (E/A) of LV relaxation [18]–[20]. LV hypertrophy is extremely common in patients with ESRD [21]. As reported by the VALIANT echo study, renal impairment was associated with larger LA volumes and increased LVMI after myocardial infarction [22]. It was also demonstrated that LAVI was strongly associated with diastolic function grade [23], [24]. We found that GFR was significantly correlated with LAVI and LVMI, as well as E/E′, deceleration time and E/A in univariate analysis. However, in multivariate analysis, GFR was only correlated with E/E′. After adjustments for potential confounding factors, patients in E/E′>15 group were still more likely to have Stage 3 CKD (defined as 30<GFR<59) compared with those in E/E′≤10 group. The present study showed that E/E′ was sensitive for detecting abnormal LV relaxation, especially in the relationship with renal function.

IVRT is also used for the direct estimation of LV relaxation^ 25^. However, IVRT by itself has limited accuracy, reductions in preload (and increases in afterload) exhibit a transmitral pattern similar to impaired relaxation (↑IVRT, ↓E, ↑DT) [25], and it is significantly related with age and heart rate [25]–[27]. In our study, the patients with E/E′>15, also with longer IVRT, who were older, with slower heart rate and higher blood pressure than patients with 10<E/E′≤15 or E/E′≤10. This fact indicated these factors might lead to a higher end diastolic pressure and impaired diastolic function in this patients group. According to the Heart Failure and Echocardiography Associations of the European Society of Cardiology, IVRT showed a variable outcome in terms of the predictive value for LV diastolic dysfunction [13]. In our study, we found that IVRT and E/E′ were correlated with GFR in univariate analysis while in multivariate analysis, only E/E′ was correlated with GFR. It suggested that E/E′ was superior to IVRT in detecting the association between LV diastolic function and renal function.

Although eGFR has been widely used in the studies investigating the relationship between LV function and renal function, the association between small reductions in eGFR and abnormalities of cardiac structure and function is unclear [28], [29]. In an analysis of 40 non-CKD patients and 202 CKD patients, LV diastolic dysfunction was observed even in patients with early stages of CKD [28]. In contrast, an independent relationship was not observed between eGFR and parameters of cardiac structure or function in an analysis of 540 hypertensive patients [29]. Variability in serum creatinine measurements makes MDRD equations substantially less accurate and GFR may be misestimated when renal function is relatively well preserved, as in our population [30]. However, radiolabeled tracers (such as ^99m^Tc-DTPA) enable accurate (low bias and high precision), and reproducible measurements of GFR, since the plasma clearance of ^99m^Tc-DTPA has been shown to correlate well with the renal clearance of inulin (r>0.90) [31]. There were significant differences in both ^99m^Tc-DTPA measured GFR and estimated GFR using the modified MDRD equation for Chinese patients among patients of E/E′>15, 10<E/E′≤15 or E/E′≤10. However, eGFR had no correlation with E/E′ or lgNT-proBNP in multivariate analysis. By using the radiolabeled tracers (^99m^Tc-DTPA) to estimating GFR, our results showed that LV diastolic function was associated with renal function in essential hypertensive patients.

Albuminuria is a marker of subclinical renal damage in hypertension and it could be a parameter or a condition that directly causes cardiovascular disease or renal dysfunction [9], [32]. To evaluate the relationship between LV diastolic dysfunction and subclinical renal damage, UACR was determined in our study. There were significant differences in UACR among patients of E/E′>15, 10<E/E′≤15 or E/E′≤10. UACR was significantly correlated with E/E′ and NT-proBNP, even after adjustment for age, gender, diabetes prevalence, use of ACE-I or ARB drugs, GFR, 24-h mean systolic and diastolic blood pressure and 24-h mean heart rate. These findings suggested that LV diastolic function was associated with subclinical renal damage in hypertension.

In addition to E/E′, NT-proBNP was correlated with GFR in univariate and multivariate analyses, consistent with the findings Van Kimmenade *et al*. [33]. It is possible that NT-proBNP is cleared passively by organs with high blood outflow, such as the kidney [34], [35]. As a result, concentrations of NT-proBNP are typically higher in patients with CKD than in those without CKD [36] and NT-proBNP levels have strong inverse correlations with eGFR [37]. The association of E/E′ and NT-proBNP with GFR indicates that patients with LV diastolic dysfunction had marked and significantly lower GFR in the presence of concomitant elevated NT-proBNP. In patients with elevated NT-proBNP, LV diastolic dysfunction was significantly associated with poorer renal function, but in patients with normal NT-proBNP, there was no relationship between LV diastolic function and renal function.

Despite the expanse of information known about cardiac structure and function in patients with ESRD [7], [8], only a few studies have focused on the relationship between mild reductions in renal function and cardiac function in early hypertension [22], [28], [29]. The mechanisms responsible for the relationship between LV diastolic function and renal function in early hypertension are not well established. Activation of the renin-angiotensin-aldosterone system (RAAS) leads to renal hypoxia, vasoconstriction, intraglomerular hypertension, glomerulosclerosis and proteinuria [38]. In addition, activation of the RAAS induces LV hypertrophy and congestive heart failure [39]. Similarly, activation of the sympathetic nervous system triggers proliferation of smooth muscle cells and adventitial fibroblasts in the vascular wall of intra-renal blood vessels [40], and also promotes the development of left ventricular hypertrophy [41]. However, the mechanisms of neuro-hormonal activation in early hypertension are still unclear. The ratio of early mitral valve flow velocity (E) divided by tissue Doppler early diastolic annular early diastolic (E′) correlates closely with LV filling pressures.

Diabetes may lead to chronic kidney disease and it can harm the kidney gradually, GFR gradually fall over a period of several years [42]. Of the 42 diabetes patients in our study, 19 patients were new-developed diabetes and 23 patients were with diabetes history. In the patients with a diabetes history, GFR was much lower than the new-developed patients (GFR: 68±24 vs. 86±18 ml/min/1.73 m^2^, *p* = 0.02, data not shown). GFR was significantly correlated with the patients had a diabetes history (*r* = −0.166, *p* = 0.02, data not shown), but had no relationship with the new-developed diabetes (*r* = −0.112, *p* = 0.11, data not shown). In all the 42 diabetes patients, GFR had no relationship with diabetes (*r* = −0.045, *p* = 0.54). This result might be due to the high percentage of new-onset diabetes in our study.

Abnormalities of pressure load characterizing early hypertension may result in both LV diastolic dysfunction and increased glomerular pressure and permeability, with resultant albuminuria [43]. Albuminuria is not only a marker of subclinical renal damage, but a marker of more generalized endothelial dysfunction [44]. In our study, we found an increase of albuminuria with matched reductions in GFR, associated with increasing in LV filling pressure. These findings suggested concomitant abnormalities of cardiac, renal, and endothelial function in early hypertension.

A number of limitations of this analysis should be noted. First, since the present study is a cross-sectional study, we should observe the predictive value of E/E′ and NT-proBNP for renal dysfunction in a follow-up study. Furthermore, the current study was designed to assess associations between LV diastolic function and renal function but does not elucidate information on the mechanisms of this association.

### Conclusions

We confirmed that E/E′ was sensitive for detecting abnormal LV relaxation, especially in the relationship with renal function. Plasma NT-proBNP may be a potential biomarker, both in early LV diastolic dysfunction and renal dysfunction. LV diastolic function, assessed with E/E′ and NT-proBNP was associated with renal function in essential hypertension. Elevated E/E′ was associated with Stage 3 CKD and the presence of stage 3 CKD would inform the clinician to evaluate for the presence of LV diastolic dysfunction.
